# Epicardial adipose tissue evaluated with cardiac magnetic resonance in normal subject and in patients with ischemic and dilated cardiomyopathies

**DOI:** 10.1186/1532-429X-18-S1-P89

**Published:** 2016-01-27

**Authors:** Francesco Secchi, Andrea Cozzi, Daniel Zambelli, Marcello Petrini, Paola M Cannao, Francesco Sardanelli, Massimo Lombardi

**Affiliations:** grid.419557.b0000000417667370Radiology, IRCCS Policlinico San Donato, Milan, Italy

## Background

Epicardial adipose tissue (EAT) is considered a marker of cardiovascular disease because of its pro-inflammatory properties. The aim was to find differences of amount of EAT in three groups of patients evaluated with cardiac magnetic resonance (CMR): 1) negative CMR; 2) patients with coronary artery disease (CAD); 3) patients with dilated cardiomyopathy (DCM).

## Methods

We retrospectively evaluated 150 patients who underwent CMR (1.5 T, Siemens) in a time span of 22 months: 50 negative (mean age ± standard deviation 47 ± 12.2 years), 50 with CAD (65 ± 9.7 years) and 50 with DCM (56 ± 12.8 years). For each patient we segmented manually the EAT in short-axis cine images at end-diastolic phase with Syngo-Argus software. Volume of EAT was converted into g (g=0.9196*volume). Intra and inter-reader reproducibility in a sub-group of 30 randomly selected patients (10 negative, 10 with CAD, 10 with DCM) was tested. Mann Whitney U and Bland-Altman test were used.

## Results

Mean EAT in negative, CAD and DCM patients was 18.17 ± 10 mL, 35.07 ± 8 mL and 29.76 ± 13 mL respectively. A significant correlation was found comparing negative patients vs CAD (p < 0.001) and DCM (p < 0.001). No correlation was found comparing CAD vs DCM (p = 0.890). Overall intra and inter-reader reproducibility was up to 83% and 76%, respectively.

## Conclusions

A significant difference of EAT amount between patients with CAD or DCM and negative patients was shown, adding more evidence to a correlation between EAT and the presence/absence ischemic or dilated cardiomyopathies.

